# Stability of the Fungal Pigment from *Scytalidium cuboideum* Carried in Food-Grade Natural Oils

**DOI:** 10.3390/jof8030276

**Published:** 2022-03-09

**Authors:** Eric Hinsch, Sarath M. Vega Gutierrez, R. C. Van Court, Hsiou-Lien Chen, Seri C. Robinson

**Affiliations:** 1 Department of Wood Science & Engineering, Oregon State University, Corvallis, OR 97331, USA; emhinsch70@gmail.com (E.H.); sarathth@yahoo.co.uk (S.M.V.G.); ray.vancourt@oregonstate.edu (R.C.V.C.); 2 College of Business, Oregon State University, Corvallis, OR 97331, USA; hsiou-lien.chen@oregonstate.edu

**Keywords:** spalting, fungal pigments, natural colorants, natural oils, *Scytalidium cuboideum*, FTIR, SEM, natural crystals

## Abstract

Wood-staining fungal pigments have shown potential use as colorants for wood and textiles, with organic solvents as the pigment carrier. Natural oils have been suggested as an environmentally friendly and more available carrier; however, oils promoted color degradation. The current study examined the mechanism of said degradation and tested therapeutic and food-grade oils (instead of finishing oils) for their potential to carry draconin red, the pigment from *Scytalidium cuboideum*, without color loss over time. FTIR analysis from finishing oils indicated that oxidation was not likely the cause of color loss as the pigment could not be distinguished from the oils in the IR spectra. SEM was employed to determine if crystal degradation was contributing to color loss and indicated, surprisingly, that the crystals of draconin red formed rather than degraded over time. This suggested crystal breakdown was also not likely the cause of color loss. The pigment did not show degradation in hemp oil, flaxseed oil, and cold-pressed linseed oil when treated with β-carotene. Further in-depth chemical studies are needed to determine the mechanism of color loss in pigmented natural oils; however, food-grade oils appear to be a promising alternative to carry draconin red, without degradation of the color.

## 1. Introduction

Synthetic dyes are the most commonly used dyes in the textile industry [1]. However, these dyes have several major problems. Most are toxic or carcinogenic in at least one state of preparation or require potentially hazardous chemicals to adhere to fabrics [1]. Additionally, most synthetic dyes are fiber specific. Acid reactive dyes will dye only protein fibers. Fiber-reactive dyes will dye only cellulosic fibers. Disperse dyes will dye only manufactured fibers, such as polyester. All these dye categories require heat in at least one step of their preparation, and most require the fabric to be dyed in a heated solution, all of which require energy usage.

Natural dyes, in contrast, have been used to color textiles for thousands of years. Woad (*Isatis tinctoria* L.), which produces a blue dye, was used as far back as ancient Egypt to dye mummy wrappings [2]. It was also one of the three major dyes, along with weld and madder, used in the textile dyeing industry in medieval Europe [2]. Madder (*Rubia tinctorum* L.), which produces a red dye, was used in India in the 4th century to dye cotton [3]. The cochineal insect (*Dactylopius coccus* Costa, 1835), which produces red cochineal dye, was used by the ancient Aztecs and Mayans to dye cotton blankets and to dye woolen goods during the colonial period [4]. Mollusks, such as *Bolinus brandaris* (Linnaeus 1758), which produces the Tyrian purple used by the ancient Greeks, have also been used to dye textiles [5]. However, even natural dyes from plants require mordants, metal salts used to fix dyes, some of which contain heavy metals. Insect dyes also work best on natural fibers that have been treated with a mordant.

The pigments produced by some lichens as secondary metabolites have also been used as textile dyes for thousands of years. Reds and purples were the most common colors of lichen pigments used for textile dyeing [6,7]. These pigments were much easier and cheaper to obtain than the Tyrian purple murex dyes and had better colorfastness. Therefore, lichen dyes were used in combination with other natural dyes to enhance the colors of the other dyes. Modern investigations into ancient textiles have shown that lichen dyes were used in varying capacities through the Industrial Revolution [6,7]. In the late 19th century, the use of lichen dyes began to decline and were no longer in use in the textile industry by the mid-20th century. Today, lichen dyeing is limited almost exclusively to handcrafters and artists [8,9,10].

As with lichen dyes, dyes from fungi tend to have low colorfastness (resistance to fading). There have been multiple studies investigating the colorfastness of extracted fungal pigments as textile dyes over the last couple of decades [11,12,13,14,15,16,17,18,19,20]. The results of most of these studies have been inconsistent whether or not mordants were used. Numerous studies did find potential for fungal dyes in textiles. Several examples are:

- Anthraquinones from the fungus *Democybe sanguinea* used on polyester turned the fabric brown-orange and wine-red [11];
- Anthraquinones from *Fusarium oxisporum* dyed wool yellow and orange [13];
- *Sclerotinia* sp. dyed cotton yarn a wide variety of colors depending on mordant used [14].

Most of these studies, with the exception of [18,19,20], used the extracellular pigments from common mold fungi, such as those from *Monascus ruber* Tiegh 1884 [12] or *Penicillium* spp. [15]. The lack of colorfastness for these pigments is not surprising as they are water-soluble and are not intended to persist on substrates in nature [21]. For fungal pigments to be potentially colorfast, the pigments would need to be non-water soluble. This assertion is supported by the increased lightfastness of woad and madder when compared to weld, due to the presence of the insoluble colorant indigotin in the former two [22]. In addition, natural dyes (water-soluble) are rarely colorfast unless combined with a mordant to form an insoluble dye lake [23]. An ideal alternative to synthetic dyes and natural dyes requiring a mordant would have the ability to dye across fiber genera, be colorfast across genera, and would require no heat or additional chemicals other than the carrier.

Wood-staining fungal pigments may be able to meet these conditions. Pigments from *Chlorociboria* spp. (Seaver ex Ramamurthi, Korf and L.R. Batra), *Scytalidium cuboideum* ((Sacc. and Ellis) Sigler and Kang), and *S*. *ganodermophtherum* (Kang, Sigler, Y.W. Lee and S.H. Yun), are quite persistent in nature and demonstrate reasonably good colorfastness on textiles [18,19,20,24]. Until very recently, these pigments were extracted and carried in dichloromethane (DCM) as DCM has been demonstrated to be the most effective solvent when using wood-staining fungal pigments as a colorant [25]. However, DCM is a potential carcinogen, mutagen, and known greenhouse gas [26]. A less potentially hazardous carrier is needed for these pigments to be utilized by consumers. Numerous attempts have been made to carry these spalting pigments in less toxic solvents, none of which have had success across all three major colors [27]. This has led to the investigation into using oils as carriers for the pigments [28,29], as natural oils have historic uses in textiles, especially in waterproofing applications. However, loss of color has been seen in these tested oils, with polymerization and/or oxidation hypothesized to be the cause of the observed color loss in the previously tested oils.

The purposes of this study were twofold. The first was to understand the process by which the red pigment draconin decolors in natural finishing oils. The second was to seek alternative solvents in food-based oils. If oils can be found that do not degrade the red color produced by *S. cuboideum*, the pigment (a crystal named ‘dramada’) may be able to move from laboratory curiosity to industrial colorant—offering the world an easy to use, lightfast, stable red that does not require water, heat, or mordants to use [30]. 

## 2. Materials and Methods

### 2.1. Fungal Growth and Pigment Extraction

Fungi were grown and the pigment was harvested following the procedures in Robinson et al. [12]. Briefly, *Scytalidium cuboideum* strain UAMH 11517 (isolated from *Quercus* sp. in Memphis, TN, USA) was cultured in woodchip amended 2% malt agar media. After two weeks the plates were air-dried, shredded, and the pigment extracted using DCM. Extracts were standardized using a Konica Minolta Chroma Meter CR-5 (Konica Minolta Business Solutions U.S.A., Inc., Ramsey, NJ, USA), on the Liquid (transmittance) setting, utilizing the CIE2000 L*a*b* color space. The target color reading for *S. cuboideum* and representing 100% concentration of the pigments was L* = 82.32 ± 2.00, a* = 26.84 ± 2.00, b* = 13.19 ± 2.00 as established by Robinson et al. [31] and used in subsequent publications by the same lab.

### 2.2. Oil Saturation

The dramada pigment carrying capacity was tested for selected oils, using previously developed protocols [28]. Tested oils included 100% organic cold-pressed hemp seed oil (Canada Hemp Foods, Gibsons, BC, Canada), 100% cold-pressed unrefined flaxseed oil (NOW), 100% solvent extracted, refined, bleached, deodorized, and winterized grapeseed oil (Nature’s Oil, Aurora, OH, USA), 100% expeller expressed, refined jojoba oil (Nature’s Oil, Aurora, OH, USA), 100% expeller expressed, unrefined jojoba oil (Nature’s Oil, Aurora, OH, USA), 100% pure olive oil (Member’s Mark, multiple locations, USA), 100% MCT oil from coconut/palm kernel oil (PipingRock Ronkonkoma, NY, USA), and refined mineral oil (Nature’s Oil, Aurora, OH, USA). 

Two milliliters of oil were added to a 20 mL wide-mouth glass scintillation vial. One milliliter of lab standardized fungal pigment draconin red in DCM was added to the vial. The DCM was evaporated and the solution was left to sit overnight in a fume hood. The solution was then inspected for complete solubilization. If pigment was bound to the glass, precipitated in the bottom of the vial, or clumped together, carrying capacity was considered to have been exceeded. Any oil with a carrying capacity of less than 200% ± 2% (2:1 ratio) was eliminated from further testing. For those oils that were not eliminated, an additional 1 mL of solubilized pigment was added until carrying capacity was reached. Carrying capacity was then refined by subtracting 1 mL from the amount of pigment at which the carrying capacity was exceeded and added back in 0.5 mL increments until carry capacity was reached. The process was repeated again to confirm accuracy, using starting amounts of the refined carrying capacities minus 0.5 mL. The pigment was then added at milliliter increments. The final carrying capacity or saturation point was determined by subtracting 0.25 mL from the amount of pigment added at which the carrying capacity was exceeded. Due to the quantity of pigment needed to color even 100 mL of the oils, only the three oils with the highest carrying capacity, plus cold-pressed linseed oil (Gamblin, purchased from Dick Blick Art Materials, Highland Park, IL, USA) which had been used in previous work [28] were selected for further testing. 

### 2.3. Oil Preparation

To prepare the oils for oxidation testing, 100 mL of each oil was added to a 1000 mL glass beaker. Six hundred milliliters of draconin red solubilized in DCM was added to the beaker. A 7.0 mm × 24.5 mm octagonal magnetic stir bar was added, and the beaker was placed on a stir plate under a fume hood and stirred at approximately 230 rpm overnight to allow the DCM to evaporate and the pigment to migrate into the oils. This process was repeated once more for a total of 1200 mL of solubilized pigment to 100 mL of oil. The pigmented oils were divided as follows: 50 mL in a 200 mL glass beaker, and two 100 mL beakers with 25 mL of oil each. From the 50 mL portion, 1.5 mL of each was added to separate VWR (VWR, Radnor, PA, USA) polystyrene macro cuvettes (4.5 mL capacity) with a Gilson Pipetman P1000 pipette in replicates of 15. The cuvettes were capped, placed back in their divided Styrofoam storage containers, and placed in a drawer where they were stored at ambient lab conditions (~25 °C and 35% RH).

To one of the 25 mL portions of each oil, approximately 2.5 µL (~100 ppm [32]) D-α-tocopherol (≥97%) (purchased from VWR) was added. Small (3 mm × 12 mm) stir bars were added to the beakers. The beakers were placed on stir plates under a fume hood and stirred at approximately 230 rpm for 30 min. One and one-half milliliters of each pigmented, treated oil was added to separate cuvettes, in replicates of 15, and stored as above. The treating process was repeated on the second 25 mL portion of each oil with approximately 0.008 g (~30 ppm) [33] β-carotene (≥99%). Fifteen additional cuvettes were prepared with each of the remaining untreated pigmented oils. Five hundred microliters of Stoddard solvent was added to the cuvettes on top of the oils. The cuvettes were capped and stored as described above.

Separate 25 mL quantities of each unpigmented oil were prepared with α-tocopherol and β-carotene as above. These were divided into five cuvettes in 1.5 mL quantities. Ten additional cuvettes were prepared with 1.5 mL of each untreated unpigmented oil. To five of these was added 0.5 mL Stoddard solvent. The cuvettes were capped and stored as above.

Due to cracking of the tops of the cuvettes, apparently caused by the oils, a double layer of yellow pressure-sensitive VWR laboratory tape was applied to the outside of each cuvette just below the cap. 

### 2.4. Color Reading

Color loss (ΔE) was chosen as the primary output parameter due to the history of its use in spalting studies. Utilizing the same output enables comparisons across the literature. To monitor color loss, color readings were taken on a Konica Minolta Chroma Meter CR-5, on the Liquid setting, utilizing the CIE2000 L*a*b* color space. A random cuvette of each pigmented oil/treatment combination was read and set as the target to calculate ΔE*00, the overall color change in each sample compared to the target. Color readings of all 10 replicates of each pigmented oil/treatment combination prepared for color reading were taken at 24 h, 48 h, 72 h, 7 days, 14 days, 30 days, 45 days, and 60 days. Color loss was determined by ΔE*00, calculated by the Konica Minolta SpectraMagic NX CMS-S100w 2.70.0006 software. A ΔE*00 of 2.2 ± 0.2, a change visible to the human eye [34], and/or a statistically significant ΔE*00 were considered a reaction over time.

For color loss, statistical analysis was conducted with SAS for Windows ver. 9.4 TS Level 1M3. The ΔE*00 calculated by the Konica Minolta SpectraMagic NX CMS-S100w 2.70.0006 software for each pigmented/oil treatment combination was used for statistical analysis. The effects of categorical time, treatment, and their interaction on ΔE*00 were tested using a linear mixed model. Autocorrelation within individual cuvettes was modeled using a power spatial covariance structure for an unequally spaced time series. Pairwise comparisons of the eight time groups within each treatment with a Tukey adjustment were calculated from this model. Analyses were done separately for each pigmented oil. The model used was

*yij* = *pi* + *nj* + *eij* (1)

where *yij* is the overall color change (ΔE*00), *pi* is the elapsed time in hours or days, *nj* is the treatment (control, α-tocopherol, β-carotene, Stoddard solvent), and *eij* is an error term. The resultant mean ΔE*00 were studied for both statistically significant differences and ΔE*00 values greater than 2.2 ± 0.2, indicating a visually detectable change in color. The same model was used for the analysis of ΔL (change in lightness), ΔC (change in chroma), and ΔH (change in hue) to determine which factors affected ΔE*00. Assumptions of normality and constant variance were checked graphically using the model residuals and were reasonably met.

### 2.5. FTIR

Samples were prepared using the potassium bromide (KBr) method to eliminate air, as established in Guillén and Cabo [35]. FTIR grade potassium bromide powder (Alfa Aesar, Haverhill, MA, USA) was placed in a glass petri dish and dried, uncovered, for 24 h at 103 °C in a ZenithLabo Vacuum Dry Oven, model DFZ-6020 (ZenithLabo, Pamona, CA, USA).

Approximately 0.88 g of KBr were transferred to a 60 mL outer-glazed mortar and pestle and ground to break up any lumps. Twenty microliters of a prepared sample were added to the KBr with a Gilson Pipetman P20 pipette. The sample was mixed into the KBr with a stainless-steel spatula until a fine powder resulted. A 3 mm internal diameter stainless steel ThermoFisher Scientific (ThermoFisher Scientific, Waltham, MA, USA) collar was placed on a 3 mm ThermoFisher Scientific die set. Enough of the KBr/sample mixture to fill the collar was transferred. The top of the die set was placed over the collar and the die was pressed for 20 s with a ThermoFisher Scientific Handi-Press (discontinued) (personal communication). The collar and resultant pellet were removed and placed on a glass plate to be transferred to the FTIR. This process was repeated for each oil/pigment/treatment combination. Each sample was placed in the KBr sample compartment of a Nicolet iS50 FT-IR (ThermoFisher Scientific, Waltham, MA, USA). The samples were scanned at 4 cm^−1^ resolution for 32 cycles on the absorbance setting, using KBr as the background. The resultant spectra were plotted by the ThermoFisher Scientific Inc. OMNIC 9.298 software. The data set of each spectrum was examined with LabCognition ifAnalyze-RAMalyze 4.0.19.0. This process was repeated for each oil/pigment/treatment combination at the initiation of the study and again at 14 days, 30 days, 45 days, and 60 days.

FTIR data from each oil/pigment/treatment combination was examined with LabCognition irAnalyze-RAMalyze for a shift in band frequencies as described in Guillén and Cabo [36]. The 10 bands that were prevalent in most or all of the oils tested by Guillén and Cabo were selected for study. In this study, FITR was used as a qualitative tool so statistical analysis was not performed on this data. Using the LabCognition irAnalyze-RAMalyze software, the control spectrum of each unpigmented oil was overlaid with the pigmented spectrum of the same oil, and the Shift Spectrum function was used to bring them into as close alignment as possible. The overlaid spectra were then compared to determine if the pigment could be detected as a distinct band from the oils.

### 2.6. Light Microscopy

Light microscopy was employed first to determine if crystals could be detected in the oils pigmented with draconin red. A drop of each pigmented oil was placed on a VWR glass slide with a disposable plastic transfer pipette and covered with a VWR #1 glass coverslip. The slides were examined, and images were taken with a Nikon Eclipse Ni-U light microscope equipped with a Nikon DA-Ri2 camera (Nikon Instruments Inc., Melville, NY, USA). The examination was conducted at 4×, 10×, and 20×, and images were taken with the resolution that showed the most detail for each sample.

### 2.7. SEM

For SEM analysis, twelve 10 mm × 10 mm squares of white spun polyester (Dacron 54) were cut from AATCC (American Association of Textile Chemists and Colorists, Research Triangle Park, NC, USA) multi-fabric test strips. The squares were placed on a glass plate, and approximately 1 mL of newly-made pigmented oil was applied to individual squares with a plastic disposable transfer pipette. The squares were turned front-to-back two to three times with stainless steel forceps to ensure coating. Four additional squares were coated with the unpigmented oils, as controls, in the same manner. The remaining four squares were coated with the pigmented oils that had undergone the 60-day color reading analysis. The coated squares were transferred to paper toweling with forceps and the excess oil was allowed to leach out for 10 days.

Following a modified procedure outlined in Vega Gutierrez and Robinson [37], Ted Pella, Inc. (Ted Pella, Inc., Redding, CA, USA) 1/2” double-coated carbon conductive tape was applied to Ted Pella, Inc. 1/2” inch slotted head aluminum specimen mounts with a 1/8” pin. The sample specimens were cut down to 10 mm × 5 mm and placed on the tape with slight pressure for adherence. The edges of the samples were coated with Ted Pella, Inc. PELCO colloidal graphite to seal the edges to increase the conductivity of electrons in the samples and to reduce image artifacts. The graphite was allowed to dry, and the samples were then coated with gold–palladium (Au-Pd) with a Cressignton (Cressington Scientific Instrument Ltd., Walford, UK) Sputter Coater 108 Auto at 0.015 mBar with a sputtering time of 35 s for a deposition of 2.5 Å/s. The Au-Pd is used to increase the conductivity of a relatively non-conductive sample.

After coating, the samples on the specimen mounts were transferred to the stage of an FEI (Field Electron and Ion Company, Hillsboro, OR, USA) Quanta 600 F environmental SEM. Images of all samples were captured with the FEI software designed for the Quanta 600 F ver.4.1.10.2127 at both high vacuum and low vacuum with water vapor, with a beam intensity ranging from 2 kV to 5 kV and a spot size of 2 and 2.5. Combinations of these parameters were used to obtain the best images possible. For higher resolution images, the samples were transferred to an FEI Helios 650 Ultra Resolution Dual Beam FEG SEM. The beam voltage was set at 2.00 kV and the immersion mode was used for all oils except for linseed on which the free field mode was used oil due to charging.

Magnification was varied to obtain the best possible images.

## 3. Results

### 3.1. Oil Saturation 

Saturation results for draconin red across all tested oils can be found in Table S1. Previous publications noted a 2:1 ratio of solubilized pigment to oil to be the minimum saturation level [38]. With this benchmark in mind, the lowest saturation amount of draconin red in any oil (that exceeded the 2:1 ration limit) was 15.75 mL ± 0.00 mL for golden jojoba oil. The highest saturation amount was 1643.89 mL ± 0.56 mL for hemp seed oil. Hemp seed oil, flaxseed oil, and MCT oil were used, as they were the most likely to impart color to a substrate when applied due to the heavy concentration of pigment.

An interesting effect developed between oils with a high unsaturated fatty acid composition (shown in Table S2) and a high level of pigment saturation, most likely due to the higher number of active binding sites of the polyunsaturated fatty acids. Of the four oils that had the highest saturation level (hemp seed oil, flaxseed oil, MCT oil, and grapeseed oil), three (hemp seed oil, flaxseed oil, and grapeseed oil) had fatty acid compositions of greater than 85%, with the highest percentage being polyunsaturated. These results were not statistically significant but are of visual interest. 

MCT oil, being a blend of refined palm kernel oil and refined coconut oil (both of which have a relatively high percentage of saturated fatty acids), was in the top three in terms of carrying capacity for the red pigment. Why MCT oil exhibited such a high level of pigment saturation is unknown, although it may have to do with the component oils being refined. For comparison, clear (refined) jojoba oil exhibited a higher pigment saturation level than golden (unrefined) jojoba oil (approximately 11:1 and 8:1 solubilized pigment/oil ratios, respectively). The refining of plant-based oils, especially bleaching, decreases both naturally occurring α-tocopherol and phenolic acid content, leaving the oils more susceptible to oxidation [39]. As with polyunsaturated fatty acids, susceptibility to oxidation may also indicate a higher likelihood of available sites for the fungal pigments to bind.

### 3.2. Color Loss

Significant color loss was seen in most oil/additive combinations (Table 1). α-tocopherol slowed the color change in both pigmented flaxseed oil and hemp seed oil, and β-carotene prevented color change. In contrast, for cold-pressed linseed oil, the Stoddard solvent slowed the color change when compared to oil treated with α-tocopherol and β-carotene, but untreated oil had the best color stability. Pigmented MCT oil did not demonstrate any color stability.

**Figure 1:**
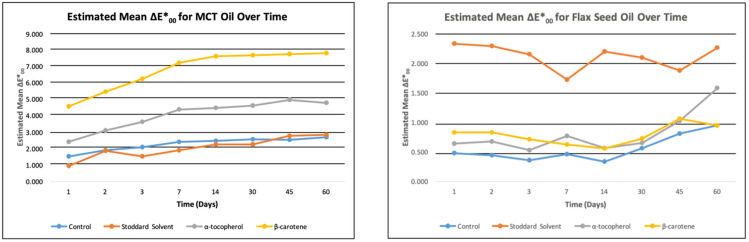
Color change over time for Flaxseed and MCT oil. Standard error = 0.193.

Although all visible overall color differences were statistically significant, not all significant differences resulted in a visible color difference, or a ΔE*00 greater than 2.2 ± 0.2 [34]. For flaxseed oil, the first significant color difference occurred as early as 24 h, but visible color differences did not occur. For MCT oil, the first significant color differences occurred at 24 h, but visible color differences did not show until 7 days (Figure 1). Both hemp seed oil and linseed oil had significant color differences, but no visible color differences.

β-carotene prevented color loss, at least for 60 days (β-carotene itself added no color to the oils, as confirmed on the Color Reader), in flaxseed oil. Based on the low mean ΔE*00 at 60 days (0.486), it is unlikely a significant or visible color change would occur for many months. Both α-tocopherol and β-carotene are antioxidants that terminate the chain reactions caused by free radicals during oxidation [32,33]. If oxidation is the reason for color loss in the pigmented oils, it follows that antioxidants would slow or prevent color loss. If some factor other than oxidation is the cause for color loss, the antioxidants may be inhibiting that process as well.

As discussed with hemp seed oil, Stoddard solvent may have broken down the crystals of the pigment, causing color loss. The color loss seen with α-tocopherol may have been a concentration issue. Evans et al. [32] showed that α-tocopherol exhibited prooxidation behavior above its optimal concentration. The concentration used in the current study, ~100 ppm, was based on a study of soybean oil, so may have been too high for cold-pressed linseed oil. The same may be true for β-carotene as well. Although no visible overall color changes were seen during the 60-day test period, the highest mean ΔE*00 value was 1.364, indicating that a visible overall color change could occur if the study had been extended.

As MCT oil is composed mainly of saturated fatty acids, oxidation would be very slow as there were few highly reactive hydrogen atoms available. Therefore, it was unlikely that either of the antioxidants would have had much of an effect on MCT oil. If the concentrations of the antioxidants were too high, which is likely as oxidation would have been slow to occur, they may have exhibited prooxidation activity, increasing the rate of color change. Stoddard solvent appeared to have slowed color loss, but only slightly. This may be due to the solvent having slowed oxygen movement from the headspace in the cuvettes to the oil. As already discussed, the color changes that occurred in both untreated and treated MCT oil make the oil unsuitable to carry draconin red.

**Figure 2:**
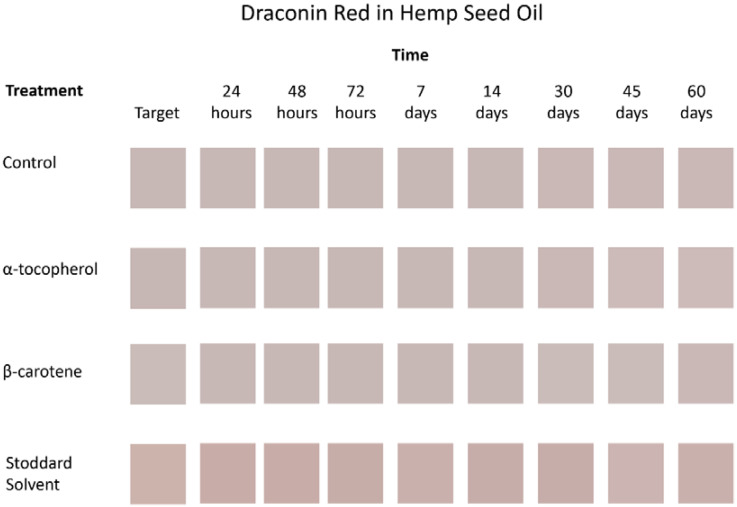
Color reproduction of draconin red in hemp seed oil for all treatments and times. Source: Adobe Photoshop CC release 19.1.3. Note: Photoshop allows for only whole integer values in the Lab color model. Colors in this figure are the closest reproductions possible with this software.

In summary, statistically significant differences do not always translate into visible color differences [38], which is showcased in Figure 2. Other variables contributed more to overall color change: ΔE*00, lightness or translucence, chroma (the purity or intensity of a color), and hue (the shade of a color; the darkness or lightness of a color when compared to a similar color). As changes in the variables can be either positive or negative, the range of the deltas over time was a more useful measure than a mean. 

Table 2 shows the range of ΔL (lightness), ΔC (chroma), and ΔH (hue) over categorical time for each oil/treatment combination. Although each factor contributed to the overall mean ΔE*00, there are some clear patterns that emerged. For 13 of the 16 combinations, ΔL was the highest or one of the highest contributors to the overall color change. In the case of the three that did not follow this pattern, flaxseed oil/Stoddard solvent, hemp seed oil/Stoddard solvent, and MCT oil/β-carotene, ΔC was the highest contributing factor. ΔH contributed very little to overall color change except for flaxseed oil/control in which it was on par with ΔL. It appears as though ΔL, a change in lightness, had the most effect on overall color change for these oil/treatment combinations, meaning the overall color change was generally a color loss, or a fading of the color rather than a change in intensity or shade.

### 3.3. FTIR

Fourier Transform Infrared Spectroscopy (FTIR) analysis was used to determine oxidation by looking for shifts in wavelength band frequencies. Pigmented oils and non-pigmented oils could not be distinguished when overlayed due to the small amount of pigment present relative to oil quantity. Oxidation of the oils was nevertheless compared, in order to understand the stability of pigment/oil mixtures and additives. Stoddard solvent and MCT oil also showed many artifacts due to being mixtures and, due to difficulty in interpretation, these samples were dropped.

As the spectra were analyzed, it was noticed that the peak at band B, 3006 cm^−1^, corresponding to cis double bond stretching vibration, was not present in any of the spectra. This band was likely overlapped by the band at 3035–3005 cm^−1^ associated with a C-H alkene stretch. As it could not be detected, band B was eliminated from further consideration. In addition, the peak at band I, 1119 cm^−1^, corresponding to saturated acyl groups, was present in only some of the spectra. As this band is inversely related to the proportion of the saturated acyl groups, which should increase during oxidation [35], and no decrease in the frequency of the band was observed, band I was eliminated from further consideration. The lack of a peak at this band or the lack of decrease in frequency was the first indication that oxidation had not taken place or was not sufficiently advanced to affect this band.

Table 3 lists the remaining bands that were examined to determine if oxidation took place (graphical representations can be found in Figures S1–S9). The wavelength bands and their respective shifts or changes are associated with stages of oxidation [36]. The time of each stage was oil-dependent but was characterized by when certain band shifts occurred. The shoulder at band F, 1630 cm^−1^, corresponding to α, β-unsaturated aldehydes and ketones [40], appeared at 30–60 days for flaxseed oil, hemp seed oil, and cold-pressed linseed oil, both pigmented and unpigmented, for all treatments. This band appears at the end of the second stage of oxidation, as determined by Guillén and Cabo [36].

Bands A, D, E, and G did not change for any treatment of pigmented or unpigmented flaxseed oil, hemp seed oil, or cold-pressed linseed oil, except for pigmented cold-pressed linseed oil with α-tocopherol in which band E decreased at 60 days. This band became a valley between peaks at 1651 and 1657 cm^−1^. The causes of this shift are unknown and may be due to foreign artifacts or human error. 

For the flaxseed oil control, band C shifted at 14 days. For flaxseed oil with β-carotene, band C was initially at 2855 cm^−1^, shifted to 2854 cm^−1^ at 14 days, then back to 2855 cm^−1^ at 30 days. For flaxseed oil with α-tocopherol, band C shifted at 30 days. For pigmented flaxseed oil with α tocopherol, band C shifted back and forth between 2854 cm^−1^ and 2855 cm^−1^, making the determination of this shift inconclusive.

For hemp seed oil with β-carotene, band H initially had two distinct peaks at 1167 cm^−1^ (peak A) and 1161 cm^−1^ (peak B). At 14 days, peak A became more intense and shifted to 1166 cm^−1^, and peak B became more rounded and shifted to 1160 cm^−1^. At 30 days, there was a single peak at 1166 cm^−1^ which then shifted to 1162 cm^−1^ at 45 days and back to 1166 cm^−1^ at 60 days.

For pigmented hemp seed oil control, band H underwent several changes. It had an initial frequency of 1167 cm^−1^, and at 14 days showed a more intense peak at 1161 cm^−1^ with a smaller peak at 1166 cm^−1^. The spectrum for 30 days was unusable in this region due to artifacts, but at 45 days the peaks reversed, 1166 cm^−1^ being more intense than 1161 cm^−1^. At 60 days, there was a single peak at 1166 cm^−1^.

For pigmented cold-pressed linseed oil control, initially, at band H, there was a peak at 1163 cm^−1^ that comprised two small shoulders at 1166 cm^−1^ and 1161 cm^−1^. At 14 days, this resolved into a single peak at 1165 cm^−1^ where it remained at 30 days. At 45 days, this peak shifted to 1167 cm^−1^, then shifted back to 1165 cm^−1^ at 60 days.

These seemingly odd shifts in band H may be due to the oil progressing from first stage (FS) oxidation to second stage (SS) oxidation as the noted shift did not occur until after FS oxidation in the study by Guillén and Cabo [36]. As this band is associated with saturated acyl groups, the observed fluctuations could be due to changing concentrations of secondary byproducts of hydroperoxide breakdown which occurs at the end of FS.

For MCT oil control, although there was no change in band E, the peak was much lower in intensity than with the other oils. This is most likely due to the high saturated fatty acid and correspondingly low oleic acid content of MCT oil. Band F appeared at 60 days and band J shifted at 60 days. For MCT oil with β-carotene, band H was initially peaked at 1165 cm^−1^ with a small shoulder at 1159 cm^−1^. At 14 days, band H resolved into a single peak at 1159 cm^−1^. At 30 days, this peak shifted to 1156 cm^−1^ and a small shoulder began to form at 1165 cm^−1^. At 45 days, the peak shift to 1158 cm^−1^ and the shoulder at 1165 cm^−1^ peaked with an intensity nearing the peak at 1158 cm^−1^. At 60 days, both peaks resolved into a single peak at 1161 cm^−1^. This was a lower frequency for band H and was most likely due to the composition of mostly saturated MCT oil as compared to the other oils which were mostly unsaturated [36]. For MCT oil with Stoddard solvent, there were not enough useable spectra to determine any band shifts except for the shift in band J at 60 days. For MCT oil with α-tocopherol, there was only one useable spectrum, so no comparisons could be made.

For pigmented MCT oil control, band E gradually decreased through the test period, and band J appeared at 60 days. Band H initially peaked at 1158 cm^−1^, then shifted to 1162 cm^−1^ at 14 days. The peak remained at 1162 cm^−1^ at 30 days, but a less intense peak appeared at 1170 cm^−1^. At 45 days, these two peaks resolved into a single peak at 1160 cm^−1^ and remained there at 60 days.

As before, the lower frequencies observed for band H are likely due to the composition of MCT oil. For pigmented MCT oil with β-carotene, the spectra for 14 days and 30 days were unusable. There was an apparent increase in intensity in band A at 45 days and 60 days, associated with first stage (FS) oxidation. Band F appeared at 45 days and band J shifted at 60 days. For pigmented MCT oil with Stoddard solvent, there was an apparent decrease in intensity of band E at 14 days, band F appeared at 60 days, band H shifted at 30 days, and band J shifted at 60 days. For MCT oil with α-tocopherol, band F appeared at 60 days, band H shifted at 60 days, and band J shifted at 60 days.

There was very slight evidence that all the oils, pigmented or unpigmented, for all treatments were in second stage oxidation by the end of the 60-day test period, based on the spectral band frequency shifts elucidated by Guillén and Cabo [36]. However, fewer than 50% of the band shifts expected were in evidence, which makes the oxidation stage or evidence of oxidation speculative, at best. Table 4 shows the possible beginning of SS oxidation for pigmented oils with each treatment compared to the first visible color change observed for the same. The only apparent relationship between these times is flaxseed oil with α-tocopherol. If SS oxidation first occurred at 14 days and the first visible color change corresponding to that oil/treatment was between 1–14 days, color loss could have been attributed to FS oxidation. However, the only instance of SS oxidation at 14 days was cold-pressed linseed oil with β-carotene which exhibited no visible color loss. Based on these comparisons, and the minute evidence of oxidation with any oil or treatment, oxidation is likely not the cause of color loss in the pigmented oils. If oxidation is occurring, based on the times that possible SS oxidation was observed with the β-carotene treatment compared to other treatments, it does not appear that β-carotene is preventing or slowing the oxidation of the oils, despite its apparent stabilizing effect on pigmented flaxseed oil and cold-pressed linseed oil. It is possible, therefore, that β-carotene is somehow stabilizing draconin red, preventing or slowing color loss in pigmented flaxseed oil and cold-pressed linseed oil, although this cannot currently be assessed due to the lack of ability to track changes of the pigment in oil with FTIR (see below).

It is also possible that a form of oxidation and/or polymerization was happening to the pigment itself. Although the oils were not undergoing oxidation, the pigment itself might be undergoing an oxidation–reduction reaction.

Hydroperoxide produced in the initial stages of oil oxidation could be reacting with the pigment rather than the oils, causing the pigment to oxidize and the oils to remain stable. In the same manner, as in oils, β-carotene could also be halting the free radical chain reaction of the pigment, which is why hemp seed oil, flaxseed oil, and cold-pressed linseed oil treated with β-carotene demonstrated color stability. The issue with possible disruptions in the alternating carbon–carbon double bonds of the pigment remains and could be affected by a redox reaction in the pigment itself. The behavior of draconin red is still a mystery. It seems the more learned about the pigment, the more questions about its interactions and reactions arise. A more in-depth chemical analysis would need to be performed on pigments in oils over time to determine precisely what is happening at a molecular level.

### 3.4. Microscopy

**Figure 3:**
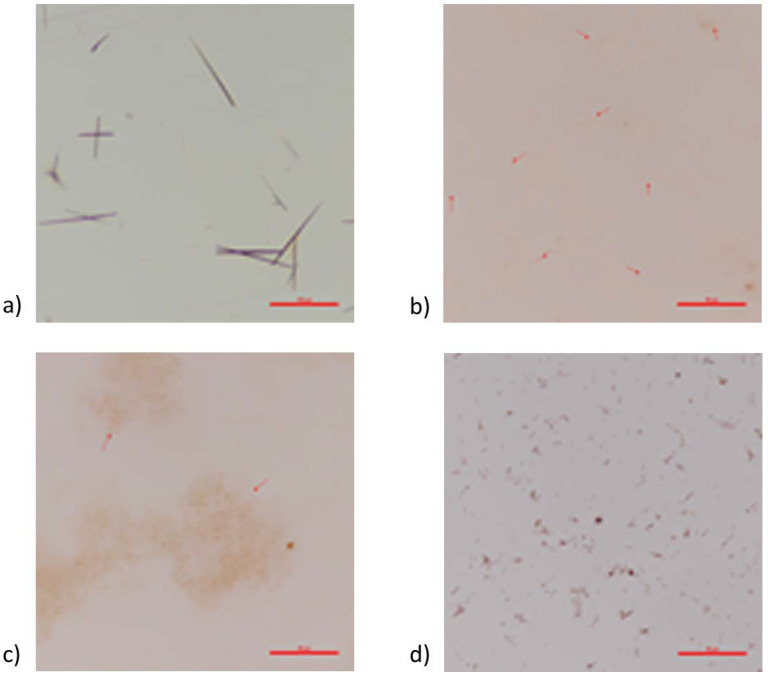
Light microscopy images for pigmented oils. (**a**) Hemp seed oil (scale bar = 100 µm); (**b**) flaxseed oil (red arrows point to crystals, scale bar = 50 µm); (**c**) cold-pressed linseed oil (red arrows point to crystals, scale bar = 50 µm); (**d**) MCT oil (scale bar = 50 µm).

Crystals were found to be present in pigmented oils (Figure 3). Pigmented hemp seed oil showed crystals over 50 µm in size, with smaller ones in pigmented flaxseed oil. The crystals were smaller and aggregated in pigmented linseed oil. There was also some flocculence in the linseed oil which may be due to the high saturation of the oil with pigment. In pigmented MCT oil, there were readily evident medium-sized crystals.

**Figure 4:**
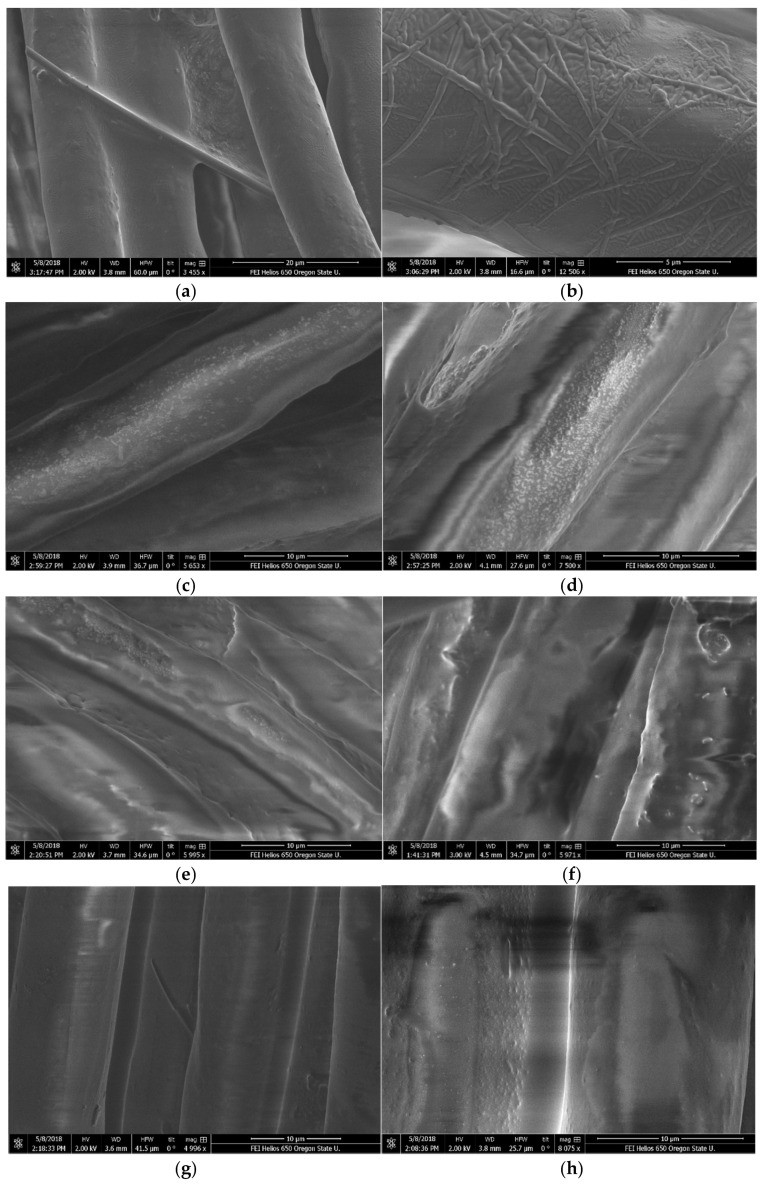
SEM images of freshly pigmented oils on spun polyester, taken with Helios 650 SEM HV = 200. (**a**) Fresh pigmented hemp seed oil; (**b**) 60-day pigmented hemp seed oil; (**c**) Fresh pigmented flaxseed oil; (**d**) 60-day pigmented flaxseed oil; (**e**) Fresh pigmented linseed oil; (**f**) 60-day pigmented linseed oil; (**g**) Fresh pigmented MCT oil; (**h**) 60-day pigmented MCT oil.

Freshly pigmented oils and pigmented oils that went through the 60-day testing period were deposited on spun polyester and examined with scanning electron microscopes (SEM) to determine if draconin red crystals were present, if they appeared, and if they changed in size, shape, or number (Figure 4). Freshly applied pigmented hemp seed oil showed few large crystals. The crystals that did appear lay on top of the oil and fiber. In contrast, 60 day-pigmented hemp seed oil showed many large crystals both laying on top of and wrapping around the fibers under the oil coating. This indicated that they may have formed, instead of breaking down, with time. 

Flaxseed oil showed small, aggregated crystals, as was also seen in light microscopy. These appeared randomly dispersed when oil was first applied, though by the end of 60 days, the crystals formed more linear patterns—potentially the result of polymerization or aggregation. In the freshly pigmented linseed oil, clumps similar to those in flax oil can be seen. These may be the beginnings of the crystallization process. At the right of the image of 60-day pigmented linseed oil is what looks like grains of rice. As the surface of the fibers are smooth and the oil appears as amorphous clumps, the grains are likely tiny dramada crystals, as similar crystals have been seen with draconin red solubilized in ethanol. 

Near the center of the image of freshly pigmented MCT oil is a very distinct, fully-formed dramada crystal. By contrast, the image of 60-day pigmented MCT oil shows very small granules resembling sandpaper, which may be either amorphous formations from the oil or pigment which has been degraded into smaller crystals, either by oxidation or polymerization.

Based on the low number, small size, and/or lack of linear organization displayed by the crystals in freshly pigmented oils under SEM, it would appear that the oils are solubilizing the crystals. However, under the light microscope, numerous crystals can be seen in all four oils. With DCM as the carrier, numerous crystals wrapped around the polyester fibers. It is likely the crystals are interacting with the oils in combination with the polyester fibers. Interestingly, in hemp seed oil the crystals appeared to form under the oil and remained in contact with the polyester fiber. Previous studies have shown that draconin red dyes polyester very well. This was attributed to the crystals having a preferential affinity for polyester [19,20].

In this study, the authors hypothesize that the flax oil showed the beginning of crystal formation. After 60 days, the random patterns of small particles could be seen to form more linear patterns, perhaps as a precursor to crystal formation. In linseed oil, the crystals also appeared to form under the layer of oil, although they were so small it was difficult to tell if they were wrapping around the fibers. In the cases of these three oils, instead of breaking down, the crystals began to form, perhaps due to an interaction with the polyester. The reason for the crystal formation is unclear. It may be that particles of draconin red are attracted to each other and pull together over time resulting in crystal formation, as clumping has been observed with the pigment solubilized in ethanol. As the pigment is non-polar, hydrogen bonding or Van der Wal’s forces may be the reasons for pigment particle attraction. Discovering the reason for crystal formation is a possible avenue for further study. 

MCT Oil was the only oil in which the expected observations were made. The crystals, plainly visible in freshly pigmented oil, broke down into small granules. This may be due to the precipitate in MCT oil noted earlier. It also may be due to the high saturated fatty acid content of the oil, that this oil was the only refined oil that was tested, or that this oil was a mixture of oils. In future studies, SEM examination could be made of the pigment in refined and unrefined palm kernel oil and refined and unrefined coconut oil to elucidate if there are different behaviors of the crystals in the different media that comprise MCT oil.

As crystal formation, rather than degradation, was observed even in oils that showed a visible color loss (flaxseed oil and linseed oil), degradation of the crystal structure was determined to not be the cause of color loss (with perhaps the exception of MCT oil). Further studies on behavior, structure, solubility, and affinity of the dramada crystals would need to be conducted to draw any conclusions about the relationship between the crystals and color loss of draconin red in oils.

Hemp seed oil, untreated and treated with α-tocopherol or β-carotene, both antioxidants, exhibited no visible color loss over the 60-day study period. The reason for this stability is unknown. Flaxseed oil and cold-pressed linseed oil treated with β-carotene also exhibited no color loss over the study period. These three oils have the potential to act as carriers for draconin red, which would make the pigment safer to use and more available to those outside the scientific community. FTIR analysis of both treated and untreated pigmented and treated and untreated unpigmented oils showed only very slight evidence that oxidation occurred, and comparisons with the timing of color loss indicated that oxidation was not the cause of color loss in the oils. The microscopic analysis did not yield significantly interesting visual clues. Induced oxidation of the pigmented oils may help clarify if oil oxidation is playing a role in color loss. 

SEM analysis showed that, except for MCT oil, dramada crystals formed over time in the oils rather than breaking down. Therefore, the breakdown of the crystalline structure of draconin red is also not the cause of color loss in the oils. It is possible that color loss may be a result of the interaction of the pigment with the oils or some form of degradation of the pigment itself resulting in the interruption of the alternating carbon–carbon double bond naphthoquinone structure, such as a redox reaction initiated by the first stages of oil oxidation. Further in-depth chemical analysis in different carriers over time would need to be conducted to determine the precise mechanism of color loss and elucidate ways to prevent it.

## 4. Conclusions

Draconin red, being a secondary metabolite of wood-staining fungi and therefore a natural, biological substance, rarely behaves as expected. There is still many opportunities for research on draconin red, and wood-staining fungal pigments in general. Opportunities exist not only in applied mycology but also in chemistry, crystallography, value-added wood products, and textiles. Collaborative efforts in these fields may help to unravel the mysteries inherent in these enigmas of nature. Results from this research indicated that while oxidation is unlikely the cause of the red color degrading in natural oils, β-carotene significantly stabilizes the color, and may be the key to future industrial adoption of draconin red as a textile colorant.

## Figures and Tables

**Table 1:** Times at which tested oils showed significant color change (ΔE*00), as determined by Tukey-adjusted simple effects comparisons. Significance was determined at the *p* < 0.05 level.

Oil Type	Unadjusted	Stoddard Solvent	α-Tocopherol	β-Carotene
Flaxseed oil	72 h	24 h	45 days	No sig. change
Hemp seed oil	60 days	7 days	60 days	No sig. change
MCT oil	24 h	24 h	24 h	24 h
Cold-pressed linseed oil	No sig. change	7 days	72 h	72 h

**Table 2:** Range of ΔL, ΔC, and ΔH over time for all oil/treatment combinations.

Oil	Treatment	Range of Mean ΔL	Range of Mean ΔC	Range of Mean ΔH
Flaxseed	Control	1.019	0.600	1.066
Flaxseed	Stoddard solvent	1.530	2.740	0.540
Flaxseed	α-tocopherol	1.811	1.271	0.197
Flaxseed	β-carotene	0.382	0.292	0.107
Hemp seed	Control	0.652	0.782	0.266
Hemp seed	Stoddard solvent	1.513	1.995	0.461
Hemp seed	α-tocopherol	1.250	0.644	0.282
Hemp seed	β-carotene	1.187	0.624	0.319
Linseed	Control	0.913	0.521	0.289
Linseed	Stoddard solvent	1.207	0.874	0.183
Linseed	α-tocopherol	1.141	0.349	0.188
Linseed	β-carotene	0.502	0.410	0.219
MCT	Control	0.915	0.995	0.381
MCT	Stoddard solvent	1.803	1.494	0.372
MCT	α-tocopherol	2.315	2.301	0.908
MCT	β-carotene	1.763	3.017	0.738

Red highlight indicates highest contributors to ΔE*00 with standard error of ±0.2. Green highlight indicates visible change and highest contributors to ΔE*00 with standard error of ±0.2.

**Table 3:** Remaining initial band frequencies, chemical correspondences, and expected changes with oxidation. FS = First Stage, SS = Second Stage. ^1^ [36], ^2^ [41], ^3^ [40], ^4^ [35], ^5^ [42]. * This corresponds with the band at 988 cm^−1^ which caused the weak band at 983 or 985 cm^−1^ to shift.

Band	Initial Frequency (cm^−1^)	Chemical Correspondences	Expected Change with Oxidation	Oxidation Stage
A	3467–3470	Formation of hydroperoxides	Shift to 3348, 3437, or 3558 cm^−1^ and increase in intensity	FS
C	2854	Symmetric and Asymmetric stretching of aliphatic CH2 functional groups ^2^	Slow increase to 2855 cm^−1^	FS to SS
D	1746	Ester carbonyl functional groups of triglycerides ^1^	Shift to 1743 cm^−1^	End of SS
E	1654	Carbon–carbon double bonds of cis-olefins ^1^	Decrease in intensity to disappears	End of SS
F	1630	α, β-unsaturated aldehydes and ketones ^3^	Appears	End of SS
G	1417	Rocking vibrations of CH bonds of cis-distributed Olefins ^1^	Sharp decrease to 1416 cm^−1^	After FS
H	1163	Saturated acyl groups ^4^	Shift to 1166–1167 cm^−1^	SS
J	983 or 985	Bending vibrations of CH trans, trans-conjugated olefinic double bonds ^5,^*	Shift to 988 cm^−1^	Beginning of SS

**Table 4:** Second stage oxidation compared to color loss by time for pigmented oils. Times in hours (24, 48, and 72) have been converted to days for ease of comparison. Green highlight indicates a match between oxidation times and the first visible color change.

Oil	Treatment	Time of Possible First Evidence of SS Oxidation (Days)	Time of First Visible Color Change (Days)
Flaxseed	Control	30	1
Flaxseed	β-carotene	45	N/A
Flaxseed	Stoddard Solvent	30	7
Flaxseed	α-tocopherol	60	60
Hemp seed	Control	45	N/A
Hemp seed	β-carotene	60	N/A
Hemp seed	Stoddard Solvent	45	1
Hemp seed	α-tocopherol	60	N/A
Cold-pressed linseed	Control	60	14
Cold-pressed linseed	β-carotene	14	N/A
Cold-pressed linseed	Stoddard Solvent	45	7
Cold-pressed linseed	α-tocopherol	60	1
MCT	Control	60	3
MCT	β-carotene	45	1
MCT	Stoddard Solvent	30	1
MCT	α-tocopherol	60	1

## Supplementary Materials

The following supporting information can be downloaded at https://www.mdpi.com/article/10.3390/jof8030276/s1, Table S1: Saturation results for draconin red; Table S2: Fatty acid composition of tested oils; Figure S1: FTIR spectrum of pigmented flax seed oil at the outset of the study. Source: GraphPad Prism 7; Figure S2: FTIR spectrum of pigmented flax seed oil after 14 days. This spectrum was only partially useable. Source: GraphPad Prism 7; Figure S3: FTIR spectrum of pig-mented flax seed oil after 30 days. This spectrum was only partially useable. Source: GraphPad Prism 7; Figure S4: FTIR spectrum of pigmented flax seed oil after 45 days. Source: GraphPad Prism 7; Figure S5: FTIR spectrum of pigmented flax seed oil after 60 days. Source: GraphPad Prism 7; Figure S6: Comparison spectra for pigmented (orange) and unpigmented (blue) flax seed oil. Source: LabCognition irAnalyze-RAMalyze ver. 4.0.19.0; Figure S7: Comparison spectra for pigmented (red) and unpigmented (blue) hemp seed oil. Source: LabCognition irAnalyze-RAMalyze ver. 4.0.19.0; Figure S8: Comparison spectra for pigmented (red) and unpigmented (blue) linseed oil. Source: Lab-Cognition irAnalyze-RAMalyze ver. 4.0.19.0; Figure S9: Comparison spectra for pigmented (red) and unpigmented (blue) MCT oil. Source: LabCognition irAnalyze-RAMalyze ver. 4.0.1.
