# Supplementary material for: Stability of the Fungal Pigment from Scytalidium cuboideum Carried in Food-Grade Natural Oils
Source: J Fungi (Basel). 2022 Mar 9;8(3):276. doi: 10.3390/jof8030276 (PMC8949540; doi:10.3390/jof8030276)
Supplement: Supplementary file 1 [file jof-08-00276-s001.zip › Supplementary figures.pdf]

Supplemental Figures: Sample FTIR Spectra

Note: Some of the spectra were unusable or partially useable. These spectra contained too many artifacts, either from unequal mixing of the sample with KBr or improper pressing, to be entirely useful for analysis. Those that were unusable were not used in any analyses. For those that were partially useable, only bands C, D, and H were used for frequency comparisons. Some of the initial band frequencies did not match exactly with the observations of Guillén and Cabo (1999). In these cases, adjustments were made in the expected frequency shift. For example, if the initial frequency of band C was 2855 cm^{-1}, a shift to 2856 cm^{-1} would be considered relevant (as opposed to the 2854 to 2855 cm^{-1} shift given by Guillén and Cabo). These slight differences are likely due to differences in instrument calibration

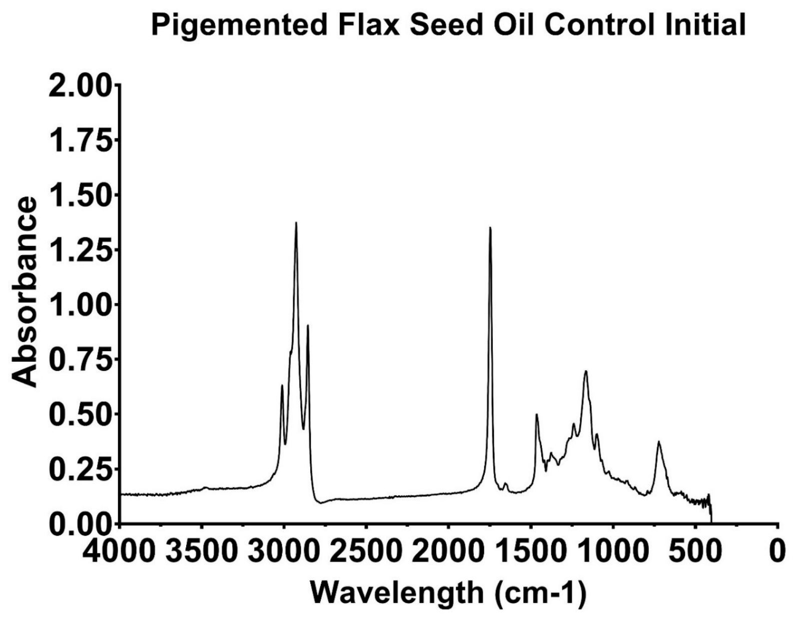

Figure S1. FTIR spectrum of pigmented flax seed oil at the outset of the study. Source: GraphPad Prism 7.

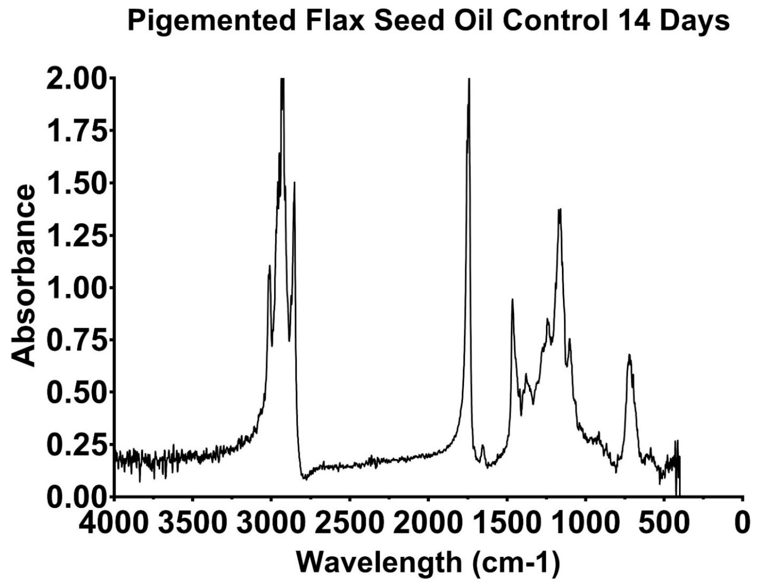

Figure S2. FTIR spectrum of pigmented flax seed oil after 14 days. This spectrum was only partially useable. Source: GraphPad Prism 7.

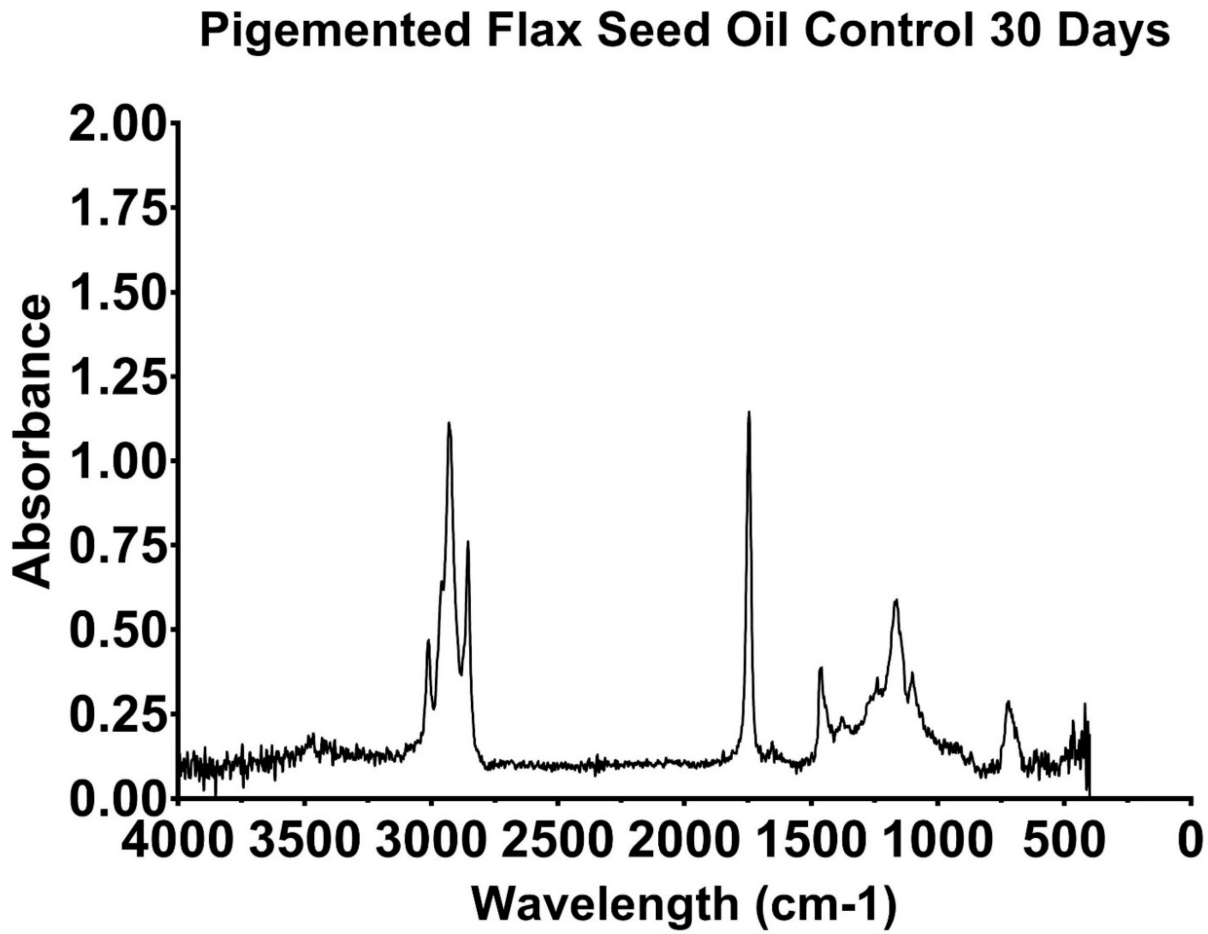

Figure S3. FTIR spectrum of pigmented flax seed oil after 30 days. This spectrum was only partially useable. Source: GraphPad Prism 7.

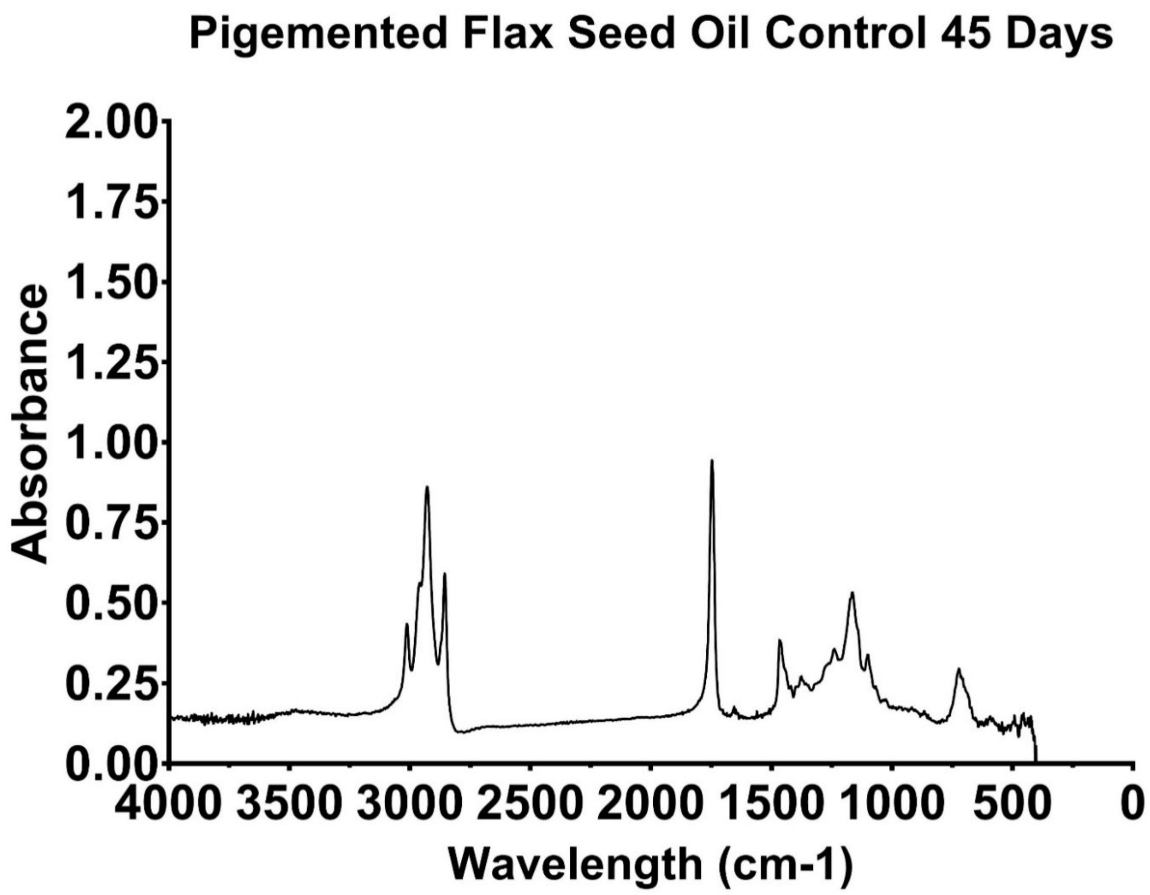

Figure S4. FTIR spectrum of pigmented flax seed oil after 45 days. Source: GraphPad Prism 7.

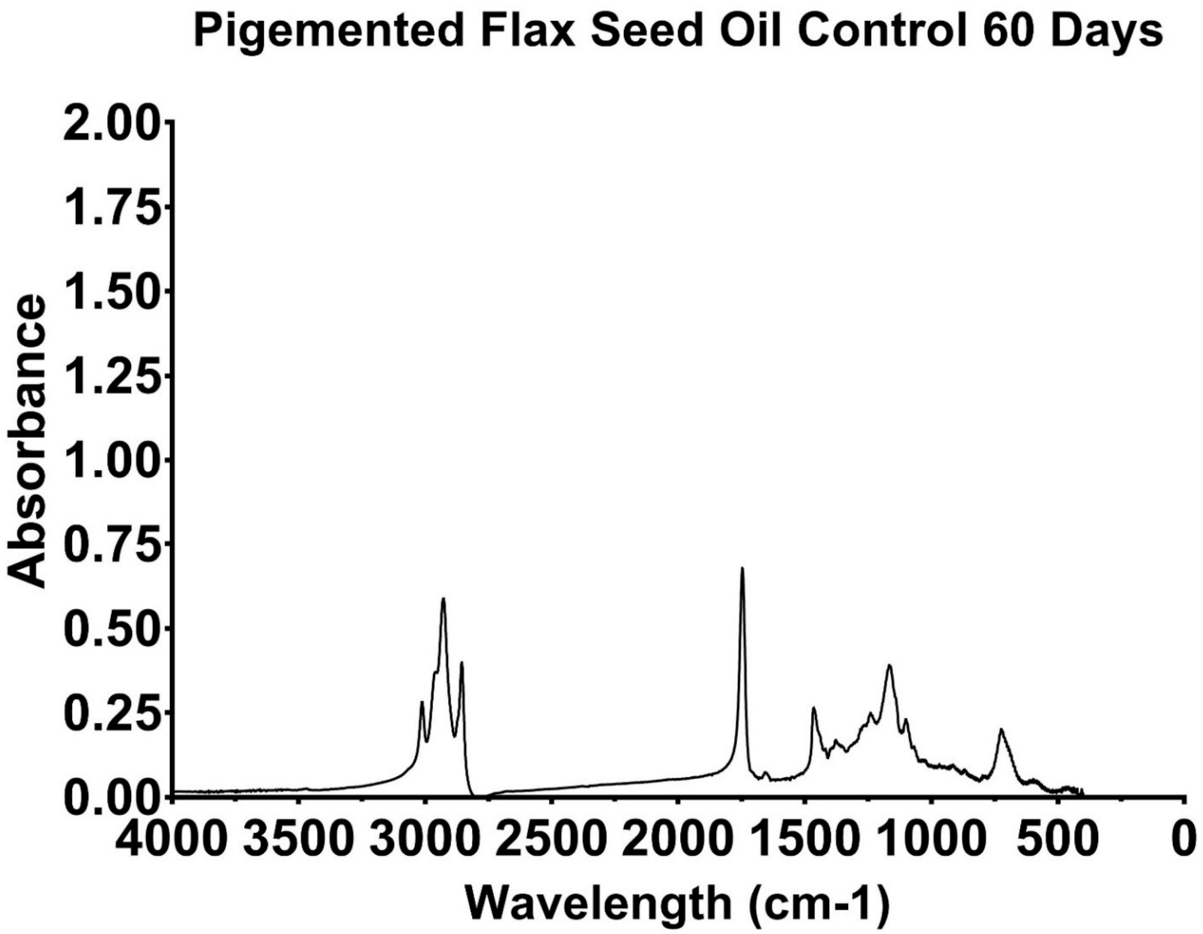

Figure S5. FTIR spectrum of pigmented flax seed oil after 60 days. Source: GraphPad Prism 7.

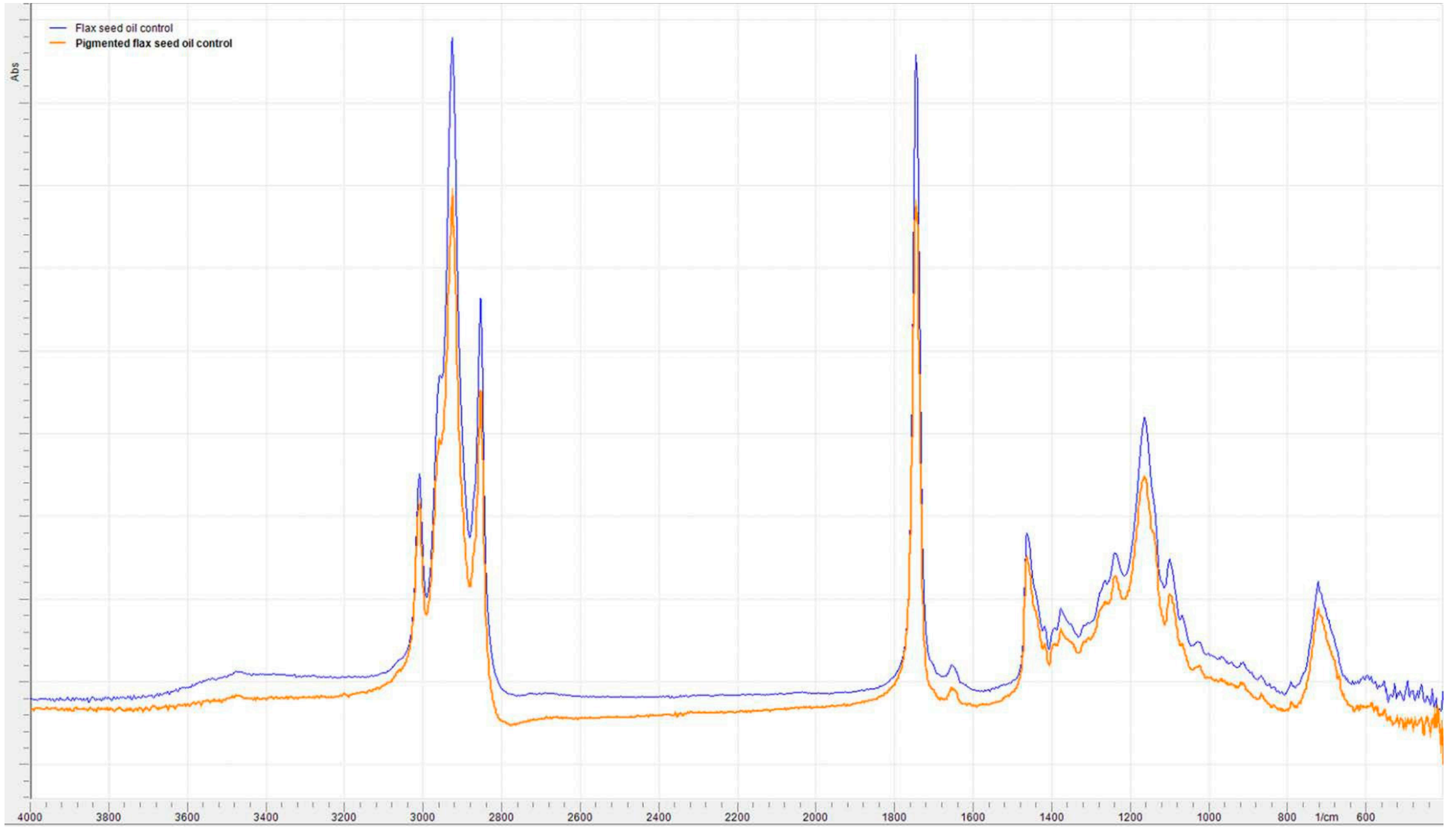

Figure S6. Comparison spectra for pigmented (orange) and unpigmented (blue) flax seed oil. Source: LabCognition irAnalyze-RAMalyze ver. 4.0.19.0.

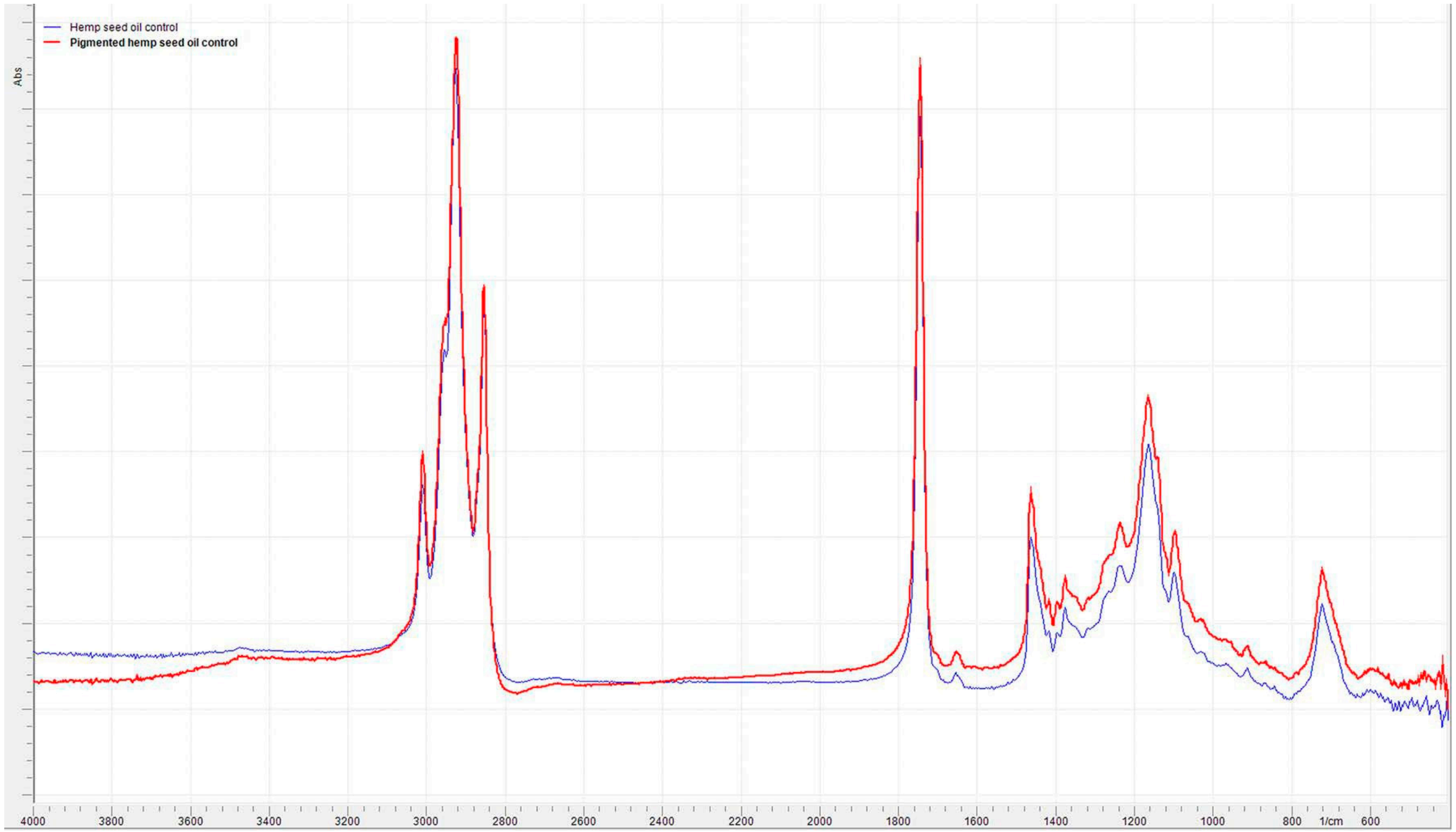

Figure S7. Comparison spectra for pigmented (red) and unpigmented (blue) hemp seed oil. Source: LabCognition irAnalyze-RAMalyze ver. 4.0.19.0.

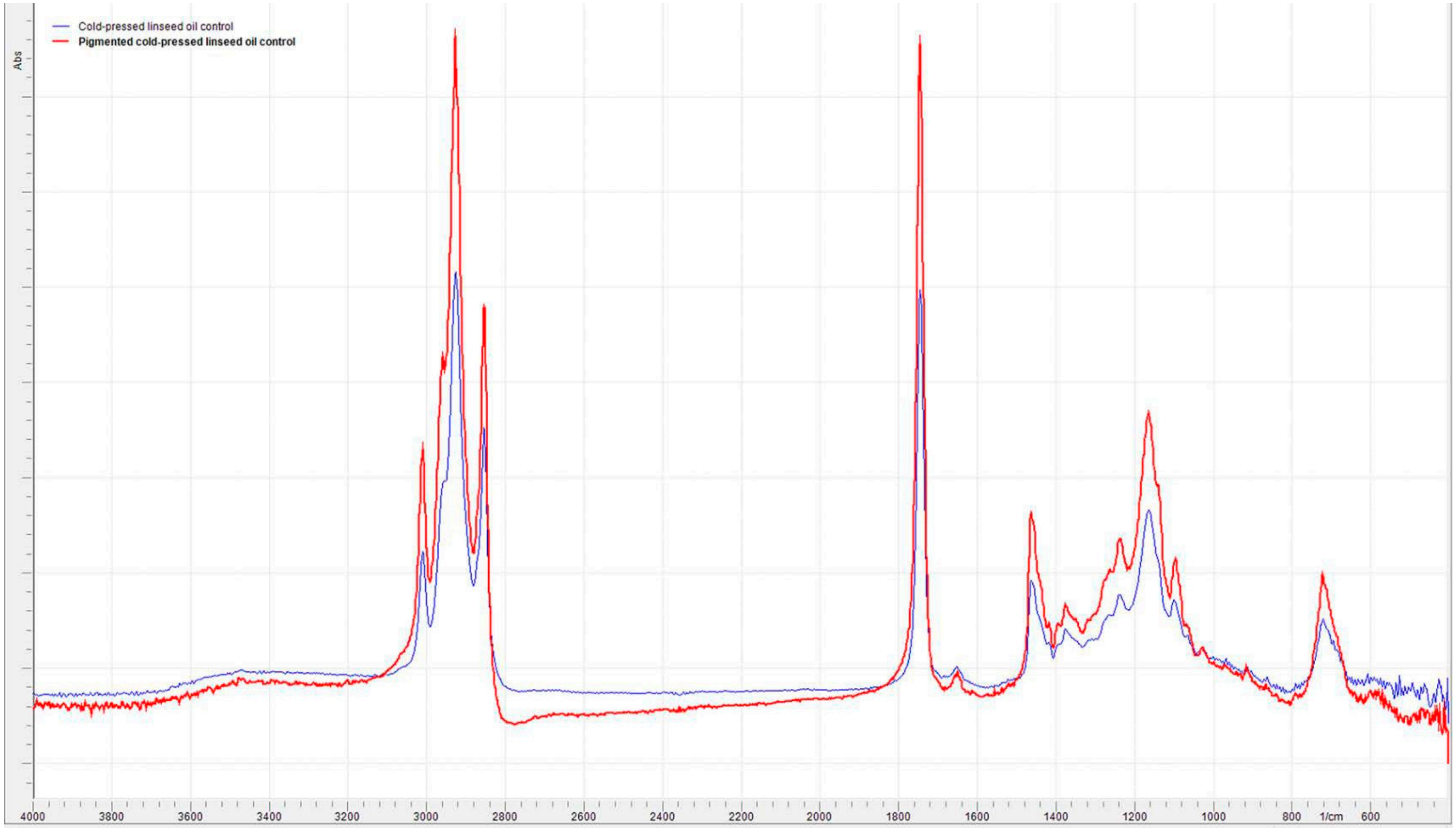

Figure S8. Comparison spectra for pigmented (red) and unpigmented (blue) linseed oil. Source: LabCognition irAnalyze-RAMalyze ver. 4.0.19.0.

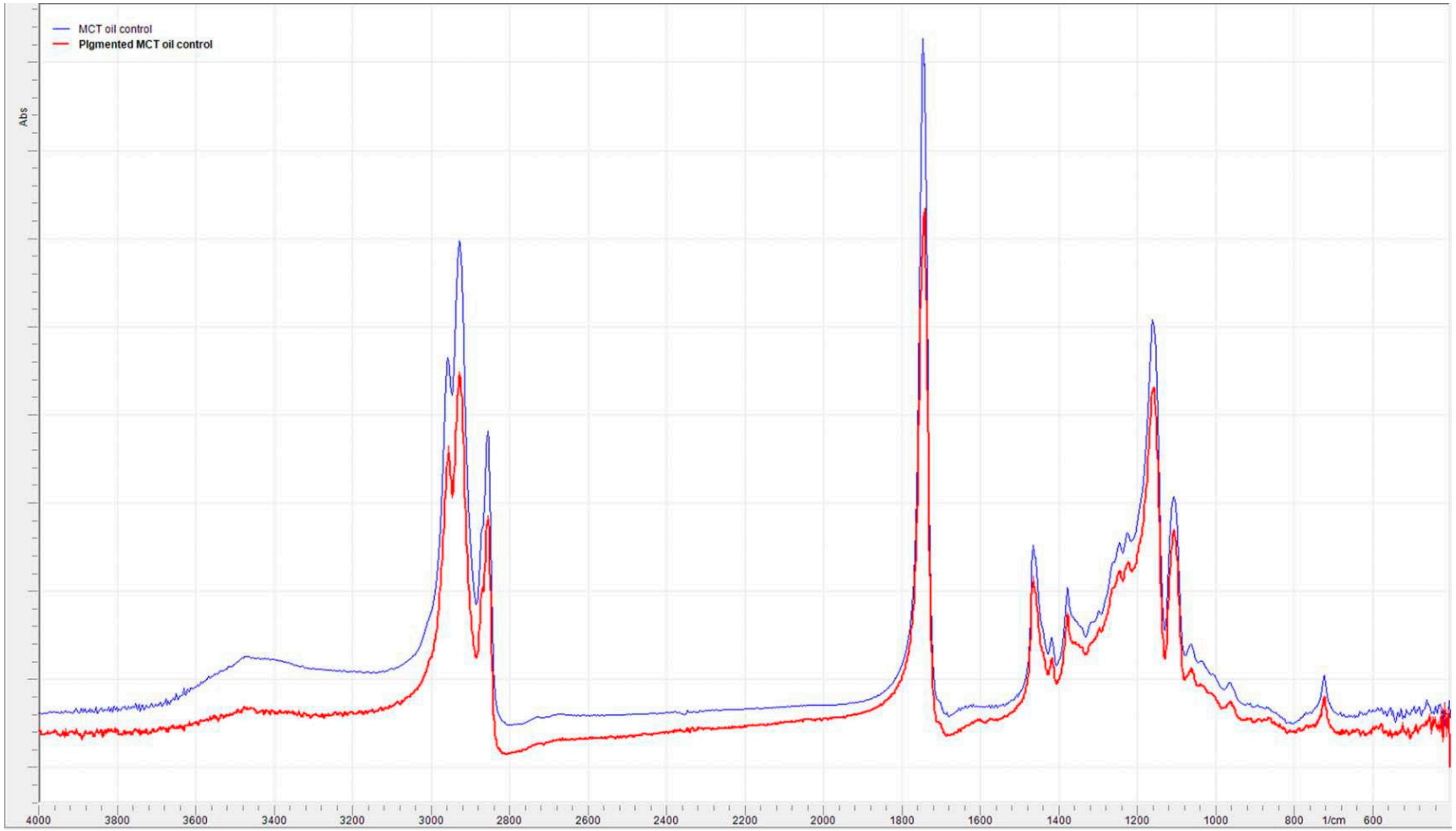

Figure S9. Comparison spectra for pigmented (red) and unpigmented (blue) MCT oil. Source: LabCognition irAnalyze-RAMalyze ver. 4.0.1
