# Supplementary material for: Stability of the Fungal Pigment from Scytalidium cuboideum Carried in Food-Grade Natural Oils
Source: J Fungi (Basel). 2022 Mar 9;8(3):276. doi: 10.3390/jof8030276 (PMC8949540; doi:10.3390/jof8030276)
Supplement: Supplementary file 1 [file jof-08-00276-s001.zip › Table S1.pdf]

Table S1. Saturation results for draconin red. All replicates for golden jojoba oil and mineral oil fell out of solution with the exact same amount of pigment.

Oil	Mean Saturation Amount of Solubilized Pigment (mL)	Standard Error	Minimum Amount of Solubilized Pigment Needed to Saturate 100 mL of Oil (mL)
Flax seed oil	32.20	0.97	1561.52
Grapeseed oil	28.70	3.25	1272.67
Hemp seed oil	33.45	0.57	1643.87
Clear jojoba oil	21.60	1.17	1021.70
Golden jojoba oil	15.75	0.00	787.50
MCT oil	28.50	2.26	1312.64
Mineral oil	4.00	0.00	200.00
Olive oil	25.55	0.53	1250.87
