# Supplementary material for: Stability of the Fungal Pigment from Scytalidium cuboideum Carried in Food-Grade Natural Oils
Source: J Fungi (Basel). 2022 Mar 9;8(3):276. doi: 10.3390/jof8030276 (PMC8949540; doi:10.3390/jof8030276)
Supplement: Supplementary file 1 [file jof-08-00276-s001.zip › Table S2.pdf]

Table S2. Fatty acid composition of tested oils. Sources: [1](United States Department of Agriculture, 2018), [2](Busson-Breysse, Farines, & Soulier, 1994). *The exact composition of MCT oil is unknown as percentages of palm kernel and coconut oils were not listed and can vary. **Mineral oil is a petroleum-based oil.

**Oil**	**% Saturated**	**% Monounsaturated**	**% Polyunsaturated**
Flax seed[1]	9.0	18.4	67.8
Grapeseed[1]	11.0	16.1	69.9
Hemp seed[1]	7.0	9.0	82.0
Clear jojoba[2]	0.9	99.1	0.0
Golden jojoba[2]	0.9	99.1	0.0
MCT*			
Palm kernel[1]	49.3	37.0	9.3
Coconut[1]	82.5	6.3	1.7
Mineral**	N/A	N/A	N/A
Olive[1]	13.8	73.0	10.5
